# Association between parental smoking and child exposure to environmental tobacco smoke in Israel

**DOI:** 10.1186/s13584-023-00585-6

**Published:** 2023-12-19

**Authors:** Tamar Berman, Efrat Rorman, Luda Groisman, Lital Keinan-Boker, Tal Shimony, Zohar Barnett-Itzhaki

**Affiliations:** 1 Public Health Services, Ministry of Health, 39 Yirmiyahu Street, Jerusalem, Israel; 2 Department of Health Promotion, School of Public Health, Sackler Faculty of Medicine, Tel Aviv University, Tel-Aviv, Israel; 3 Israel Center for Disease Control, Israel Ministry of Health, Gertner Institute, Sheba Medical Center, Tel Hashomer, Ramat Gan, Israel; 4 Faculty of Engineering, Ruppin Academic Center, Emek Hefer, Israel; 5 Research Group in Environmental and Social Sustainability, Ruppin Academic Center, Emek Hefer, Israel

**Keywords:** Environmental tobacco smoke, Secondhand smoke, Cotinine, Human biomonitoring, Children exposure, Passive smoking

## Abstract

**Background:**

Environmental tobacco smoke (ETS) exposure in children can cause delayed lung development and lifelong cardiovascular damage. The aim of this study was to measure ETS exposure in children in Israel in 2020–2021 using urinary cotinine (UC) measurements and to assess correlates of ETS exposure, including parental smoking.

**Methods:**

In the framework of the National Human Biomonitoring Program, spot urine samples and questionnaire data were collected from 166 children aged 4–12 years, during the years 2020–2021. We collected urine samples in 233 adults, 69 of whom were parents of children included in the study. Parents of participating children were asked about parental smoking, child’s exposure to ETS and smoking policy at home. Cotinine and creatinine were measured in urine. Creatinine-adjusted and unadjusted urine cotinine (UC) geometric means were calculated. Associations between potential correlates and UC concentrations were analyzed in univariate and multivariate analyses. For 69 child-parent pairs, correlation between child and parental UC was analyzed.

**Results:**

Based on urinary cotinine measurement, 65.2% of children of smokers are exposed to ETS, compared to 20.7% of children in non-smoking families. Greater numbers of smokers living in the home (beta = 1.27, *p* < 0.01), and low maternal education (beta = − 2.32, *p* < 0.01) were associated with higher levels of UC in a multivariate analysis. Spearman correlations showed a positive moderate correlation between UC in 69 child–parent pairs (r = 0.52, *p* < 0.01).

**Conclusions:**

In order to reduce child exposure to ETS, smoking parents should be urgently targeted for smoking cessation and smoke-free home interventions. Further interventions are needed to protect all children from ETS.

**Supplementary Information:**

The online version contains supplementary material available at 10.1186/s13584-023-00585-6.

## Background

The U.S. Surgeon General has determined that there is no safe level of exposure to secondhand smoke [1]. Exposure to environmental tobacco smoke (ETS), also known as secondhand smoke and passive smoking, in children causes a wide range of respiratory and development adverse effects, including sudden infant death syndrome, asthma, middle ear infections, lifelong cardiovascular effects, problems with lung development, and lifelong cardiovascular problems [1–3]. ETS exposure may be associated with cognitive deficits among children, even at extremely low levels of exposure [4].

Many countries have adopted policies to prevent non-smokers from being exposed to ETS, including bans on smoking in public places and initiatives to promote smoke-free homes. In Israel, smoking is currently prohibited in most closed public places, in schools, and in outdoor public areas such as playgrounds and zoos [5]. However, the home environment, where children and adults spend much of their time, is unregulated. In addition, there are no restrictions on smoking in cars or on balconies, or in common areas in multi-residential buildings [6, 7]. National surveys and human biomonitoring (HBM) studies in Israel indicate widespread exposure to ETS in children and non-smoking adults, with higher levels of exposure in children from low socioeconomic status (SES) backgrounds and from the Arab population [8–10].

The current study was conducted in the framework of the National Biomonitoring Program in Israel, which aims to periodically evaluate exposures of the general population (children and adults) in Israel to environmental contaminants and to measure nutritional biomarkers. We aimed to: (1) measure ETS exposure in children in Israel using UC measurements, and (2) assess correlates of ETS exposure in children in Israel, including parental smoking and children’s sociodemographic characteristics.

## Materials and methods

Spot urine samples for children aged 4–12 years and for adults were collected between July 2020 and June 2021 as part of the 1st cycle of the National Human Biomonitoring Program in Israel. Child–adult pairs were recruited when possible (agreement to participate by parent with child in appropriate age group, and agreement for child participation). The study was approved by the Tel Hashomer Hospital ethical committee (6924-20-SMC). All parents and adult participants signed informed consent forms.

We determined a “quota system” for the sample so as to represent the population distribution of urban versus rural dwelling (with urban defined as a population of more than 2000) and the two major ethnic groups in Israel (Jews and Arabs) as well as wide geographical representation. Participants were recruited via social media. Messages about the study were distributed via Facebook and Whatsapp. Participants were recruited until each “quota” was filled for that subgroup.

Urine samples were collected from 166 children and questionnaire data were obtained via telephone interview with parents by trained interviewers, using a structured questionnaire. For 69 children with a parent who participated in the National Biomonitoring Study, urine sample and questionnaire data from one parent only was collected.

### Reported measures

The interview consisted of a dietary and lifestyle questionnaire, a demographic questionnaire, and questions regarding exposure to ETS. The questionnaire was based on that used previously in the National Health and Nutrition Survey [10]. Parents of child participants were asked, “In the past month, to what extent was your child exposed to smoking of others (other people who smoked near your child)?”. Parents who answered that their child was exposed to smoking were asked whether the child was exposed to smoking at home, at school, and/or at other places (for example friends’ house, events, public areas). For adult participants, there were detailed questions on current and previous smoking, including of different tobacco products (electronic cigarettes, non-combustible cigarettes) and self-reported ETS exposure.

The questionnaires included questions on smoking policy at home, whether the participant lived in a multi-residential building, and neighbor smoking. Parents were asked which of the following statements was true regarding family policy on smoking in the home (including cigarettes, electronic cigarettes, IQOS, or nargila): (1) Smoking is permitted inside or near the house, including balconies and yards; (2) Smoking is forbidden inside the house, but allowed on balconies/yards; (3) Smoking is forbidden inside or near the house, including balconies and yards. Regarding neighbor’s smoking, parents were asked “do your neighbors (including family members living nearby) smoke”? Of note, questions on smoking policy and neighbor smoking were not included in the National Health and Nutrition Survey questionnaire and have not been validated.

Socioeconomic status was measured using total household income as a categorical variable according to grouped categories (low/medium/high). Monthly household income up to 6,500 NIS (New Israeli Shekel; 1 NIS ≈ 0.27 $US) was defined as low socioeconomic status (SES), household income between 6501 and 13,000 NIS was defined as medium, and household income above 13,000 was defined as high.

Maternal and paternal education were analyzed as categorical variables, by collapsing nine categories in the questionnaire to three categories: low (up to high school education), medium (nonacademic degree), or high (academic degree).

ETS exposure (by parental report) was analyzed as a categorical variable, by collapsing four categories in the questionnaire to two categories (yes compared to little or no).

Neighbor smoking was analyzed as a categorical variable based on frequency of smoking (frequent, moderate, sometimes, never). Smoking policy at home was also analyzed as a categorical variable (banned in all places, allowed in balcony and yard, permitted in all places).

### Collection and analysis of urinary cotinine and creatinine

On the day of the interview, children and adult participants were given a 120-ml urine specimen container. Spot urine samples were collected in the containers and maintained at below 4 °C for a maximum of 12 h until they were transported to the Ministry of Health National Biomonitoring Laboratory. Urine samples were aliquoted and frozen at – 20 °C. Aliquots were transferred to the Shamir Medical Center for measurement of creatinine.

Urinary cotinine concentrations were measured at the Ministry of Health National Biomonitoring Laboratory. Cotinine analysis was performed using the gas chromatography mass spectrometry procedure as validated and published by the German Research Foundation (DFG) working group “Analyses in biological materials” [11]. In brief, cotinine was extracted by dichloromethane from urine samples, spiked with an isotope-labeled analogue used as an internal standard. The final extract was injected to the Agilent 7000GC/MS Triple Quad Instrument and analyzed in MRM Mode.

The method followed standard quality assurance and quality control procedures. Urinary cotinine was quantified using internal standard calibration procedure and certified analytical standards. Quality control was performed by analyzing aliquots of control material in each series (each ten samples) and accuracy was validated by the annual successful participation in international proficiency test (G-EQUAS) for all parameters. Limit of quantification (LOQ) for cotinine in urine was 0.5 µg/L.

Urinary creatinine concentrations were measured at Shamir Medical Center on a COBAS 8000 autoanalyzer (Roche Diagnostics, Indianapolis, IN, USA) according to the manufacturer’s instructions.

Urinary analyte concentrations were provided in units of μg/liter. In order to correct for variable dilutions among spot samples, these concentrations were divided by urinary creatinine concentrations (g creatinine/liter urine) to generate creatinine-adjusted analyte concentrations.

Concentrations below the LOQ for cotinine were imputed using a β-substitution for left censored data [9, 12, 13]. We calculated percent of participants with UC above the LOQ, and geometric mean and median of cotinine in all participants. We conducted all calculations using both unadjusted (μg/liter) and creatinine adjusted (μg/g) values.

### Statistical methods

Since data were left-skewed and not normally distributed we used non-parametric statistical tests, or used the log value of urinary cotinine, depending on the analysis.

We compared data from our study to available data on UC concentrations in children (2015–2016) in one previous study in Israel [9] using the Mann–Whitney U test. We used population weights to adjust for differences between population distribution levels of Jews and Arabs (0.74 for Jews, 0.26 for Arabs) [9].

### Bivariable analyses

We calculated the correlation between age, number of smokers in the house, family income and UC concentrations (non-adjusted/adjusted) using the Spearman rank correlation test. We used the Mann–Whitney test to compare the UC concentrations (non-adjusted / adjusted) between genders, and between Arabs compared to Jews. We also calculated the odds ratio of having UC above the level of quantification in Arabs and Jews. The differences in UC concentrations in children, by smoking policy and neighbor smoking, were tested by using both the Mann–Whitney and Chi-square tests (referring to two levels of cotinine concentrations: low–below 0.5 μg/g and high–higher than 0.5 μg/g). Differences in home smoking policy between children from Arab and Jewish populations were compared using Z-test for comparing two proportions.

### Parent–child pairs UC comparison

In 69 parent–child pairs, we calculated the correlation between UC concentrations (creatinine non-adjusted / adjusted) using the Spearman rank correlation test. We repeated this analysis separately for children of smoking parents and children of non-smoking parents.

### Multivariable analyses

In order to assess variables associated with child exposure, we conducted a multivariable linear regression on the log of urinary cotinine. We included ethnicity, number of smokers, home smoking policy and maternal education in the analysis. We included urinary creatinine as a separate independent variable, as recommended by Barr et al. [14].

## Results

### Participants and demographics

166 children ages 4–12 years (mean age of 7.2 years) participated in the current study: 86 (52%) boys and 80 (48% girls); 144 (87%) Jewish and 22 (13%) Arabs. There were four siblings in the cohort. Of participants who answered whether the parents smoke (162/166), 46 (28%) of the children have at least one smoking parent, while 116 (72%) of the children are of nonsmoking parents. Of the 46 children with at least one parent who is a smoker, there are 37 children where both parents smoke, six children where only their father smokes, and three children where only their mother smokes (See Additional file 1: Table 1). We excluded one child from the analysis with unadjusted UC concentration of 389 µg/L as this very high urinary cotinine concentration likely indicates active smoking [15].

#### ETS exposure in children

Median creatinine adjusted concentrations in participants in the current study (0.37 µg/g) were lower than in 2015–2016 (1.7 µg/g) (*p* = 1.68e-05). Of note, 90th and 75th percentile concentrations of UC were also lower in the current study. In addition, the percent of children with UC concentrations above the LOQ (0.5 µ/L) was 33% in the current study compared to over 60% in 2015–2016. In addition, the percentages of children with UC above 1 µ/L (17.5% compared to 63.6% in 2015–2015) and above 5 µ/L (5.4% compared to 19.1%) were lower in the current study. The population weighted geometric mean creatinine adjusted cotinine concentration was 0.49 μg/g (compared to 1.6 μg/g in 2015–2016).

### Bivariable analyses

#### Urinary cotinine covariates (reported ETS exposure, parental smoking and demographic characteristics)

The percentage of children exposed to ETS based on UC measurement (33%) was consistent with the percentage of children exposed based on parental report (34%). Of note, over 10% of parents reported that their child is exposed to ETS to a very great or great extent (Table 1). UC concentration and parental reported exposure to ETS were significantly correlated (R = 0.46, *p* < 0.01). In univariate analysis, median UC concentrations were ten times higher in children of parents who reported that their children are exposed to ETS to a very great or great extent (2.69 µg/g) compared to little or none (0.25 µg/g) (*p* < 0.001).

**Table 1:** Urinary creatinine adjusted cotinine concentrations (µg/g) in children of smoking and non-smoking families

	Smoking Families (N = 46)	Nonsmoking Families (N = 119)	All (N = 165)
Percent with UC above the LOQ	65.2%	20.7%	33.3%
Mean + STD, Urinary Cotinine	3.25 ± 4.55	0.82 ± 1.93	1.52 ± 3.06
Mean + STD, Log Urinary Cotinine	0.18 ± 1.57	-1.1 ± 1.15	-0.71 ± 1.42
Median Urinary Cotinine	1.34	0.23	0.34
Geometric Mean Urinary Cotinine	1.2	0.33	0.49

STD = standard deviation

*P* value smoking compared to nonsmoking families  < 0.001

Table 1 presents urinary creatinine adjusted cotinine concentrations in children in smoking and nonsmoking families, and overall. Children of smoking parents (N = 46) had statistically significantly higher median UC concentrations (1.34 µg/g compared to 0.23 µg/g, *p* < 0.001) and higher percentages of urinary cotinine above the LOQ (65.2%) compared to children of non- smoking parents (20.7%), (chi-square *p*-value < 0.001).

There was also a statistically significant correlation between number of smokers in the house and the adjusted creatinine levels (R = 0.39, *p* < 0.01).

Children who had been in automobiles with smokers also had statistically significantly higher UC (3.49 µg/g compared to 1.29 µg/g, *p* < 0.01). All children who had been in automobiles with smokers were children of smokers except one child.

Median creatinine adjusted UC concentrations were statistically significantly higher in Arab children (0.68 µg/g) compared to Jewish children (0.28 µg/g) (*p* = 0.02) and Arab children had higher odds of having quantifiable cotinine in urine (OR = 2.82, CI = 1.13–7.0) (*p* = 0.03).

Children who lived in multi-residential buildings (N = 107) did not have higher UC than children who lived in private homes. In all children whose parents reported neighbor smoking (N = 144), frequency of neighbor smoking was statistically significantly associated with creatinine adjusted UC (Spearman correlation between frequency of smoking and UC, R = 0.2, *p* = 0.02). In children of non-smokers, neighbor smoking was statistically significantly associated with creatinine adjusted UC (Spearman correlation between frequency of neighbor smoking and UC, R = 0.38, *p* < 0.01), and also in a chi square analysis (*p* = 0.03). Of note, parental and neighbor smoking were not significantly correlated.

In all children, family income was statistically significantly negatively associated with adjusted UC (R = − 0.7, *p* = 0.005). Family income was not associated with UC in children of non-smokers. Of note, 20 of the smoking parents (43.5%) and 40 of the nonsmoking parent (34.5%) did not report their income. Maternal (R = − 0.37, *p* < 0.001) and paternal (R = − 0.25, *p* = 0.01) education were statistically significantly negatively associated with creatinine adjusted UC in children of smokers. Maternal but not paternal education was statistically significantly negatively associated with creatinine adjusted UC also in children of non-smokers (R = − 0.15, *p* = 0.02).

Of 161 parents who answered the smoking policy question, slightly over 50% reported smoking is not allowed at home including the balcony. Of the nonsmoking parents, 78 (69.0%) declared smoking is not allowed, 34 (30%) reported smoking is allowed only in balconies or in the yard, and one parent reported smoking is allowed everywhere. Of the smoking parents,eight (18.2%) reported smoking is not allowed, 28 (63.6%) reported smoking is allowed only in balconies or in the yard, and eight (18.2%) parents reported smoking is allowed everywhere.

**Fig. 1:**
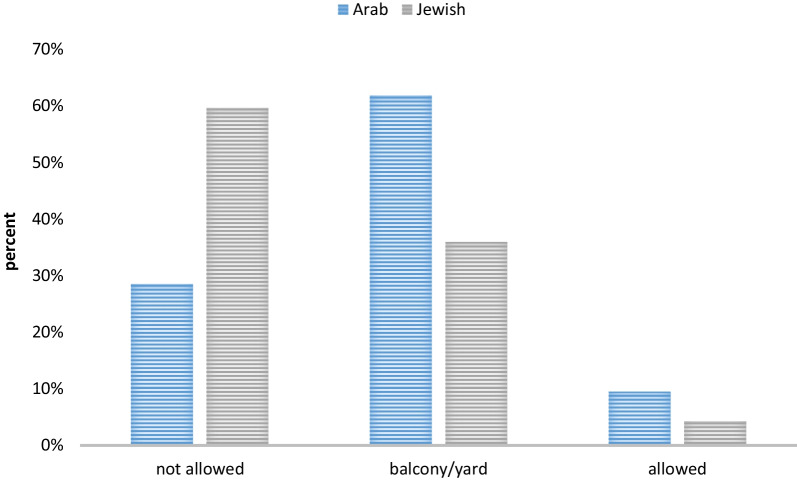
Percent of Children, by Home Smoking Policy and Ethnicity. "not allowed"—not allowed to smoke in the house, in the balcony or in the yard "balcony/yard"—not allowed to smoke in the house, but allowed to smoke in the balcony or in the yard "allowed"—allowed to smoke in any part of the house, including yard and balcony

The percentage of children living in homes where smoking is allowed everywhere was higher in children in the Arab population relative to the Jewish population but the difference was not significant (*p* = 0.41). 28.6% of the Arab parents and 59.7% of the Jewish parents reported smoking is not allowed at home, 61.9% of the Arab parents and 36.0% of the Jewish parents reported smoking is allowed in yards and/or balconies, while 9.5% of the Arab parents and 4.3% of the Jewish parents reported smoking is allowed everywhere (see Fig. 1).

In the univariable analysis which included all children, children in homes in which smoking is not allowed in all areas including balconies and yards had statistically significantly lower median UC (0.23 µg/g) than children living in homes where smoking is allowed in balconies and yards (0.58 µg/g), or homes where smoking is allowed everywhere (4.24 µg/g) (*p* < 0.05).

In the univariable analysis among children of smoking parents, only a few children lived in homes where smoking was banned in all locations. Children of smokers living in homes where smoking was allowed only in balconies or yards (1.0 µg/g) had lower UC concentrations than those living in homes where smoking was allowed everywhere (4.6 µg/g), but the difference was not statistically significant (*p* = 0.0504).

#### Parent–child pairs

For 69 of the 166 children in the current cohort there was information regarding one of their parents, including UC, creatinine and smoking status. In nine out of the 69 parent–child pairs, the parent was an active smoker. Spearman correlations showed a positive moderate correlation between the child UC and his/her parent’s UC (non adjusted: r = 0.52, *p* < 0.01, creatinine adjusted: r = 0.37, *p* = 0.002). Similar results were found in the case of pairs with non-smoking parents only (non adjusted: r = 0.35, *p* = 0.01, creatinine adjusted: r = 0.24, *p* = 0.09). Positive but not statistically significant correlations were found in the case of pairs with a smoking parent, though these correlations were not statistically significant, probably due to the small number of pairs (n = 9).

### Multivariable model

In a multivariate model including all children in the study, number of smokers (more smokers at home are associated with higher UC), and maternal education (lower maternal education associated with higher UC) were statistically significant correlates of UC concentrations (Table 2) (model significance *p* < 0.001). Home smoking policy and ethnicity contributed to the model but were not statistically significant.

**Table 2:** Results of multivariable linear regression model (n = 163)

	Unadjusted to creatinine	
	Beta coefficient	*p*-value
Intercept	− 0.4	0.95
Creatinine	1.38	0.04
Population subgroup (Arabs compared to Jews)	0.46	0.16
Number of smokers in the child’s home	1.27	< 0.01
Maternal education	− 2.32	< 0.01
Forbidden to smoke at home	− 0.27	0.29

Of note, when we added neighbor smoking to the model, this weakened the significance of the model, possible due to colinearity between variables and the fact that over 10% of parents stated that they don’t know if neighbors smoke.

A multivariable model including only children of non-smokers, which included the following variables (creatinine, ethnicity, neighbor’s smoking, maternal education, and smoking policy at home) was statistically significant (*p* =  < 0.04). The only statistically significant variable was smoking policy (beta = − 0.62, *p* < 0.01). Of note, ethnicity (Arabs compared to Jews, beta = 0.48), and maternal education (beta = − 1.35) also strongly contributed to the model.

## Discussion

Based on urinary cotinine, an objective measure of exposure to tobacco smoke, we found that 20.7% of children of nonsmokers, and 65.2% of children of smokers, were exposed to ETS. The overall proportion of exposure (32%) is low compared to our previous study in 2015/2016 (over 60%). The study was conducted during the COVID pandemic and it is possible that children’s exposure to ETS inside and outside of the home changed during the COVID pandemic as they spent less time in public areas including school, and at social events. There are conflicting reports on the impact of the COVID pandemic on potential exposure to ETS in Israel, with some surveys reporting increased home smoking [16] with others reporting reduced exposure to ETS in adults [17]. Of note, while our study took place during the COVID pandemic, participants were not recruited during lockdowns. Since we did not have data on parental smoking in the previous study, it is difficult to interpret the difference in UC in the two studies, and it is unclear if this drop reflects a real decrease in exposure to ETS in children in Israel.

We found that the variables associated with child exposure in the multivariable analysis were number of smokers in the household and maternal education. Our findings regarding the association of these variables with child ETS exposure are consistent with previous studies showing that parental smoking is the major predictor of children’s exposure to ETS [18, 19]. Our finding that UC are positively correlated in parent–child pairs (in a subset of children in the study) is consistent with previous studies showing a correlation between parental and child UC in children of smoking parents [18, 19]. Of note, we found a significant positive correlation between parent and child UC, even though most (60) of the pairs were for non-smoking parents.

The finding of the high rate of ETS exposure among children of smokers is consistent with a recent study in Israel. In 159 Jewish children ages 0–7 in smoking families, 68.8% of children in smoking families were exposed to tobacco smoke [20]. The finding of over 20% exposure in children of non-smokers is also consistent with previous research in Israel [20] and likely indicates exposure to ETS in public areas and/ or at homes of family members and friends.

Our findings and findings of previous studies underline the need for targeted interventions for parents who smoke. According to Ministry of Health data, the smoking rate in Israel among those aged 21 and over was 20.1% and was much higher in some subgroups (for example Jewish males ages 21–34, 29.8%) [21]. Others have reported smoking rate in adults ages 25–34 over 40% in some age categories (25–29, 44.7%; 30–34, 50.4%) [22].

Data are not available on the percentage of parents in Israel who smoke or percentage of children with smoking parents. In this study, over 25% of children had smoking parents; assuming this finding is generalizable to the total population of Israel, there are potentially over 750,000 children in Israel with smoking parents.

The Ministry of Health recently published a tobacco control strategy proposal, which aims to reduce smoking rates to below 5% by 2035 [23]. A complementary strategy should be developed to address the issue of ETS exposure and children’s exposure to parental smoking. In 2010, the UK government included in its national tobacco control plan the ambition to see two-thirds of households with smoking parents become smoke-free by 2020 [24]. In the USA, the Department of Health and Human Services “Healthy People 2030” program includes the target of increasing the proportion of smoke-free homes to 92.9% [25].

To date, the Ministry of Health website and baby wellness online brochures include the injunction to make the home, car, and workplace smoke-free, and instructs parents that smoking near a window or on a balcony increases exposure of children to ETS. More extensive campaigns should be considered and expanded to include non-parental care-givers. For example, campaigns in the UK have used the ‘take 7 steps out(side)’ or ‘take it right outside’ slogan to target parents who smoke at home and promote a change in their smoking behavior to create a smoke-free home [26]. The US EPA began a national smoke-free home initiative in 2001 which involved a smoke-free home pledge approach [27].

We recommend targeting parents for interventions to protect children from ETS, both through targeting them for smoking cessation and targeting them for interventions to increase distancing their smoking from their children (10 m at least) [20]. The Ministry of Health could help disseminate programs or interventions at the community, municipal, and clinical levels—once these are tested and tailored for the Israeli population. The “Smoke-Free Homes: Some Things are Better Outside” intervention in the US, an evidence-based intervention that helps households to create rules that decrease exposure to ETS, has been shown to increase home smoking bans [28]. A clinical intervention in the US showed that pediatrician advice alone may be sufficient to increase parental protection of children from ETS [29]. Of note, many studies have reported no significant reduction in child tobacco smoke exposure following interventions [30] so interventions should be carefully chosen and adapted.

The evidence regarding whether smoke-free homes is associated with lower exposure levels is not uniform. Matt found that completely smoke-free homes, in which parents smoked only in outside areas with doors and windows closed, reduced but did not eliminate child exposure [31]. Additional studies have shown that promotion of “smoke-free” programs can lead to increased home smoking bans, decreased ETS exposure, and increased cessation rates [32]. However only half of the parents, (and less than 20% of smoking parents) in the current study report having completely smoke-free homes.

Several interventions are currently being developed and tested in Israel which focus on pregnant women and their partners, and children of smokers. However, as Rosen et al*.* [33] point out, most interventions have reduced but not eliminated children’s exposure to ETS and a combination of strategies is needed to protect children from tobacco smoke in their homes. In the current study, parents received results of their children’s urinary concentrations and a brochure on reducing children’s exposure to ETS but there was no follow up study to evaluate effectiveness of interventions in this population.

It is possible that improved implementation and enforcement of laws in Israel on smoking in public places could also lead to denormalization of smoking near children. In the UK, the ban on smoking in public places and the denormalization of smoking in enclosed spaces contributed to the increase in the percentage of smoking parents adopting a policy of no smoking in the home in the UK, from 17.3% in 1998 to 75.9% in 2018 [34]. While smoking is banned in Israel in indoor public spaces and some outdoor public places where children are present (zoos and parks), oversight is lax [35]. The World Health Organization recommends ongoing monitoring of implementation of and compliance with smoke-free laws [36].

Finally, a ban on smoking in cars with children and in public areas in multi-residential buildings (stairwells, building entrances) could reduce children’s exposure to ETS, and could lead to denormalization of smoking near children. A recent study in Israel showed a large majority of respondents favored legislation and policy to limit exposure to tobacco smoke incursion in multi-unit apartment buildings, with support highest for stairwells and building entrances [6].

This study had several limitations. The most notable weakness is our sample size. Recruitment to the study was conducted via social media in both Hebrew and Arabic (Facebook and WhatsApp messages). Recruitment of children during the COVID-19 pandemic, especially of Arab children, was challenging (low response rate). The current study is based on a convenience sample, including children from a range of geographic and socioeconomic backgrounds, but the sample does not necessarily represent the Israeli population. Because we recruited the sample using social media, the sample may be biased towards higher-than-average education and income populations. If so, this might have resulted in an underestimation of the true ETS exposure, as smoking rates and ETS exposure are generally higher in lower SES populations.

Interviews were conducted by phone and not in person as planned because of social distancing requirements. In addition, parents were informed that the study was intended to measure exposure to ETS, and that they could receive their child’s individual result. In principle, knowing that the children will be objectively tested may increase the accuracy of parental report; however, this is unknown. In the question about home smoking policy, we asked about balcony/yard smoking together, without differentiating between them. Finally, the question about neighbor smoking was not validated. It is possible that people are not aware as to whether their neighbors smoke.

On the other hand, the study has strengths. We collected data on an objective measure (UC) to evaluate children’s exposure to ETS, as well as data from detailed questionnaires on children’s exposure to ETS (parental smoking, smoking policy at home, neighbor smoking).

## Conclusions

The number of smokers in the home is the major predictor of children’s exposure to ETS. 65% of children of smokers are exposed to ETS; and less than 20% of smoking parents reported having smoke-free homes. In a subset of non-smoking parents with UC data available for parent- child pairs, UC concentrations were moderately correlated.

There is an urgent need to develop national targets for reducing exposure of children to ETS in Israel [37] and for improved policies to advance the goal of zero ETS exposure in children. In order to reduce child exposure, smoking parents should be urgently targeted for smoking cessation and smoke-free home interventions. Further interventions are needed in order to protect all children from ETS exposure.

### Supplementary Information

**Additional file 1:** Characteristics of study population (children).

## Data Availability

The data underlying this article cannot be shared publicly in order to protect privacy of study participants. The anonymized data will be shared upon reasonable request to the corresponding author.

## Abbreviations

CI: Confidence interval
ETS: Environmental tobacco smoke
HBM: Human biomonitoring
LOQ: Limit of quantification
NIS: New Israeli Shekels
SES: Socioeconomic status
UC: Urinary cotinine
