# Supplementary material for: Association between parental smoking and child exposure to environmental tobacco smoke in Israel
Source: Isr J Health Policy Res. 2023 Dec 19;12:37. doi: 10.1186/s13584-023-00585-6 (PMC10731699; doi:10.1186/s13584-023-00585-6)
Supplement: Supplementary file 1 — Additional file 1: Characteristics of study population (children). [file 13584_2023_585_MOESM1_ESM.docx]

Supplementary Table 1

**Table S1: Characteristics of study population (children)**

|  | **N** | **%** |
| --- | --- | --- |
| **Age range (n=165)** |  |  |
| 4 – 8 | 107 | 65% |
| 9- 12 | 58 | 35% |
| **Population Group (n=166)** |  |  |
| Jewish | 144 | 87% |
| Arab | 22 | 13% |
| **Sex (n=166)** |  |  |
| Males | 86 | 52% |
| Females | 80 | 48% |
| **Average monthly household income (n=104)** |  |  |
| Low | 8 | 7.7% |
| Medium | 16 | 15.4% |
| High | 80 | 76.4% |
| **Parental Reported of Children's Exposure to ETS (n=161)** |  |  |
| Very great | 5 | 3.1% |
| Great | 14 | 8.7% |
| Somewhat | 38 | 23.6% |
| Not exposed | 104 | 64.6% |
| **Parental smoking status (n=162)** |  |  |
| Non smokers | 116 | 72% |
| Only father smokes | 6 | 3.7% |
| Only mother smokes | 3 | 1.9% |
| Both parents smoke | 37 | 22.8% |
| **Smoking Policy at Home (n=161)** |  |  |
| Prohibited in home including balcony and yard | 86 | 53.4% |
| Prohibited at home, allowed in yard or balcony | 62 | 39.0% |
| Smoking allowed everywhere | 9 | 5.5% |
